# Mastectomy or breast conserving surgery? Factors affecting type of surgical treatment for breast cancer – a classification tree approach

**DOI:** 10.1186/1471-2407-6-98

**Published:** 2006-04-20

**Authors:** Michael A Martin, Ramona Meyricke, Terry O'Neill, Steven Roberts

**Affiliations:** 1 School of Finance and Applied Statistics, Australian National University, Canberra ACT, 0200, Australia

## Abstract

**Background:**

A critical choice facing breast cancer patients is which surgical treatment – mastectomy or breast conserving surgery (BCS) – is most appropriate. Several studies have investigated factors that impact the type of surgery chosen, identifying features such as place of residence, age at diagnosis, tumor size, socio-economic and racial/ethnic elements as relevant. Such assessment of "propensity" is important in understanding issues such as a reported under-utilisation of BCS among women for whom such treatment was not contraindicated. Using Western Australian (WA) data, we further examine the factors associated with the type of surgical treatment for breast cancer using a classification tree approach. This approach deals naturally with complicated interactions between factors, and so allows flexible and interpretable models for treatment choice to be built that add to the current understanding of this complex decision process.

**Methods:**

Data was extracted from the WA Cancer Registry on women diagnosed with breast cancer in WA from 1990 to 2000. Subjects' treatment preferences were predicted from covariates using both classification trees and logistic regression.

**Results:**

Tumor size was the primary determinant of patient choice, subjects with tumors smaller than 20 mm in diameter preferring BCS. For subjects with tumors greater than 20 mm in diameter factors such as patient age, nodal status, and tumor histology become relevant as predictors of patient choice.

**Conclusion:**

Classification trees perform as well as logistic regression for predicting patient choice, but are much easier to interpret for clinical use. The selected tree can inform clinicians' advice to patients.

## Background

Breast cancer is a disease that affects about ten percent of Australian women. Because of its devastating impact on the community, much research has been conducted on multiple aspects of the condition, including possible causative factors, methods of treatment and patient care, and preventative measures such as breast screening. In this paper we investigate factors that affect the choice between the treatment options of mastectomy and breast conserving surgery (BCS) for Western Australian breast cancer patients. Current Australian guidelines for the treatment of early breast cancer recommend that women be treated using a multidisciplinary approach involving appropriate surgery, radiotherapy, and systematic adjuvant therapy [1,2]. The factors investigated include tumor size and histology, nodal status, martial status, aboriginality, age, method of payment, area of residence, and country of birth.

A number of studies have investigated the factors that determine the type of surgery that breast cancer patients choose [3-9]. These studies have identified a number of important factors including place of residence, age at diagnosis, tumor size, socio-economic factors, and racial/ethnic factors. Studies of this nature are important because even though there has been an increase in the use of BCS since the early 1990s, an apparent under-utilisation of BCS among women for whom such treatment was not contraindicated has been documented [10]. For this reason, research that may shed light on reasons for the underutilisation of BCS is important. For example, if it is found that women of a certain ethnicity are less likely to choose BCS when it is a viable option, education campaigns could be specifically implemented to target these women.

A recent Western Australian study found several factors that affected the likelihood of women with breast cancer receiving BCS [7]. In particular, they found that women from disadvantaged backgrounds were significantly less likely to receive BCS than those from more privileged backgrounds. In this study, the classification of an individual to a background category was based on a five category index of relative social disadvantage, with disadvantaged backgrounds and privileged backgrounds at opposite ends of this scale. However, the findings from this study were criticized because the study failed to adjust for tumor size [11]. In his criticism, Furnival stated that as a consequence of tumor size not being included in the analyses that "no reliable conclusions can be drawn as to the cause of the lower incidence of breast-conserving surgery in women from 'disadvantaged backgrounds"'. In our paper we also use data from Western Australia but address the issue raised by Furnival by including tumor size in our analyses. In addition, we employ classification trees to help elucidate the factors as well as the interaction between factors that are relevant predictors of the choice of surgery. Classification trees are gaining broader acceptance in this area of biomedical research, but they are not yet in widespread use. Our paper highlights how they can be used to improve and clarify the results obtained from the standard logistic regression approach. Classification trees enable a flow-chart to be produced that can be easily followed for a patient with a given set of characteristics in order to predict the likely treatment chosen by the patient. This property enables classification trees, unlike logistic regression models, to be readily interpreted by people with little statistical knowledge. Attempting to explain treatment decisions based on a set of patient characteristics has been termed the "propensity score method" in the literature. Graf (1997) provides an application of this method to the treatment decision of mastectomy versus BCS [12].

## Methods

Data for the study was sourced from linked administrative data obtained from the Western Australian (WA) Department of Health Record Linkage unit. The dataset was extracted from the WA Linked Database, a dynamic linkage system linking three core data sources: the WA Cancer Registry (WACR), the WA Hospital Morbidity Database (WAHMD) and the WA Death Register. The WAHMD contains comprehensive patient demographic, diagnosis and procedure information for each hospital admission occurring in any WA hospital. The dataset used consists of the linked hospital, death and WACR records containing the diagnosis, subsequent admissions to hospital and death (where applicable) of 2713 women diagnosed with one primary breast tumor in WA between 1 January, 1990 and 31 December, 1999. The WA Linked Database is unique in the Australian context and is an extraordinarily rich and comprehensive resource [13].

In this analysis, treatment was defined as the last surgical treatment within four months (120 days) of diagnosis. The date of diagnosis is defined for this study as the time at which the subject enters the study as recorded on the WACR as none of the databases linked by the Western Australian unit specifies diagnosis date. It is common for people who initially choose BCS to be readmitted for mastectomy because initial surgery reveals that the cancer is more progressed than initially advised. Thus, if a subject had a lumpectomy but was later readmitted for a mastectomy within 120 days of diagnosis, their treatment would be defined for this study as mastectomy. Individuals who had no surgery within 120 days following diagnosis were removed from the dataset. The treatment (surgery) variable was assigned as follows: a value of 0 was assigned to those subjects who received mastectomy as surgical treatment for the breast cancer within 120 days as indicated on the WACR; a value of 1 was assigned to those subjects who received BCS as the last surgical treatment for the breast cancer within 120 days as indicated on the WACR.

The explanatory variables included in the analysis were tumor size (diameter at greatest extent measured in millimetres), nodal status (the number of lymph nodes to which the cancer had spread), tumor histology (ductal, lobular, or other, for example, tubular), age of the subject in years, area of residence (metropolitan, rural or remote), the subject's country of birth, marital status, whether or not they were Aboriginal or Torres Strait Islander, and the method of payment (private or public). Table 1 contains summary information for each of these variables. The extracted data constituted 2713 patients. Patients with tumors of size greater than 100 millimetres in diameter were removed from the study. This was done to avoid these patients having an undue influence on the analysis and because there is a possibility that some of the largest recorded tumor sizes were the result of recording errors. Moreover, an important consideration is that patients with very large tumors (e.g. > 100 mm) may not even be candidates for breast-conserving surgery. There were a total of 16 patients with recorded tumor sizes greater than 100 millimetres.

Classification trees and multivariate logistic regression were used to investigate whether the variables described in the previous paragraph affected the likelihood of a breast cancer patient choosing BCS as their treatment. Multivariate logistic regression has been used in a number of studies of this nature and will be not be described further here. Classification trees are another, less pervasive, method that can be used to discriminate for a categorical response based on several, possibly interacting covariates [14,15]. In this context, classification trees create subgroups of the data with the property that within the subgroups the outcomes (choice of BCS or mastectomy) are as homogenous as possible, and between subgroups the outcomes are heterogeneous. These subgroups are created by a recursive series of rules or binary splits. For example, if we were only interested in how tumor size and area of residence were related to choice of surgery a classification tree might first split on "tumor size smaller than 20 mm", and then for such tumor sizes it might further split on "area of residence metropolitan". A classification tree of this form would be interpreted as follows: a patient with a tumor size exceeding 20 mm would be predicted to choose mastectomy; a patient with tumor size less than 20 mm and having a metropolitan area of residence would be predicted to choose BCS; and a patient with tumor size less than 20 mm and a rural or remote area of residence would be predicted to choose mastectomy. This tree has stratified the population of breast cancer patients into strata of treatment choice based on tumor size and area of residence. This simple example illustrates the high interpretability of classification trees and highlights the reason why tree representations are popular with medical scientists and doctors [15].

One potential problem with classification trees is that if continuos variables are used the tree may select "odd" splits for these variables; see, for example, Altman et al. (1994) [16]. For example, a tree model may choose to split on the continuous variables size and age at a tumor size of 27.4 mm and at an age of 61.6 years, highly specific values that are objectively meaningless. One way of avoiding this problem is to *a priori *select a few sensible splits for the continuous variables of interest. This was the approach taken in this study. Patient age and nodal status were split into three categories ≤ 40,40–60,≥ 60 years and 0,1–3,> 3 nodes, respectively, splits that have been used in previous studies [17-19]. Tumor size was split into the three size categories consistent with the American Joint Committee on Cancer and the International Union Against Cancer TNM staging system: ≤ 20 mm (T1), 20–50 mm (T2), ≥ 50 mm (T3). Creating these categorical variables out of the three continuous variables ensures that the tree can only split at sensible cutpoints, that is, at points recognised as clinically important by the medical profession. For ease of comparison, these new categorical variables were also used in the logistic regression analysis. While such a choice inherently involves information loss, the practical loss of information is slight, especially considering the increased interpretability of the resultant models.

The classification trees in this paper were fit using the rpart package available in the statistical package S-Plus [20]. The tree-fitting process initially proceeds by finding the covariate that "best" divides the subjects into two groups. The "best" split is defined as the one that results in most homogeneous subgroups with respect to the response variable, homogeneity assessed with respect to standard measures of goodness-of-fit such as the drop in deviance or the misclassification rate at each potential split. The process then partitions the subjects into the two resulting groups and repeats the splitting process in each of the two groups, a process referred to as recursive partitioning. The tree is "grown" in this way until some minimum group size is reached. This initial tree is usually too large and complicated to lead to useful inferences in the same way as an initial logistic regression fit often contains too many variables, many of them ultimately insignificant. The initial, full tree is then "pruned" to produce a simpler, more interpretable tree that adequately models patient choice of surgical treatment, compared with the full tree which likely overfits the data. Pruning a classification tree is the name given to the process of simplifying the initial tree by removing some of the lower splits. The pruning used in this paper was based on minimizing the cross-validated misclassification error across competing sub-trees. The minimum group size used and further details on the pruning process used are described below. After a suitable pruned tree is identified, a process called "burling" (examining alternative splits at each node in the tree) is used to assess the reliability of the selected tree.

## Results

An initial logistic regression model was fit to assess the relationship between surgical treatment choice and the covariates used in this study. The initial model included only main effects, and the results are presented in Table 2. The model fit indicated that tumor size was, by far, the most significant variable in the model, with subjects with T2 and T3 sized tumors choosing mastectomy more often than subjects with T1 sized tumors. Other significant variables included nodal status (subjects with nodal involvement choosing mastectomy more often than subjects with no nodal involvement), histology (subjects with lobular tumors choosing mastectomy more often than subjects with ductal tumors and subjects with "other" (mixed ductal/lobular, tubular, etc.) tumors choosing BCS more often than subjects with ductal tumors), area of residence (rural-based subjects choosing mastectomy more often than metropolitan-based subjects), and subject age (subjects 60 years and over choosing mastectomy more often than subjects aged under 60 years). The predicted importance of tumor size agrees with current clinical practice guidelines that recommend that BCS only be used for people with early stage breast cancer, where a cosmetically agreeable result is possible. For large tumors in certain areas of the breast a cosmetically agreeable result is typically not possible.

Of course, the initial model fit ignored potential interactions between variables in the model. A stepwise procedure was used to fit a larger logistic regression model that included relevant two-way interactions terms. The results of that model revealed that several interaction terms were, indeed, significant, including interactions between size and area, size and marital status, age and country of birth, age and tumor histology, age and marital status, and area of residence and country of birth. The large number of significant two-way interactions in the fitted model makes presentation of results, as well as their subsequent interpretation rather difficult. The presence of numerous significant interactions not only makes interpretation of the model coefficients difficult, it also renders the model useless as a basis for forming clinical guidelines for women faced with the choice between mastectomy and BCS.

**Figure 1:**
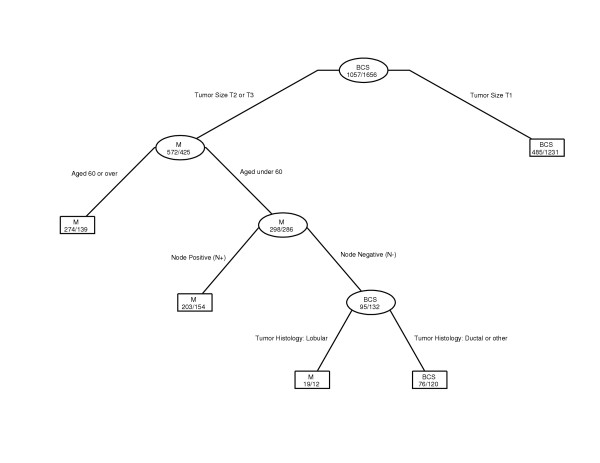
Classification tree for choice of surgical treatment for breast cancer based on the Western Australian database. In the figure, M denotes mastectomy, BCS breast-conserving surgery. Terminal nodes in the tree are represented using rectangular boxes, while non-terminal nodes are presented as ellipses. At each node, the treatment preferred by the majority of subjects at that node is printed within the node along with a numerical breakdown of how many subjects chose each treatment. For example, a node with the notation "BCS" and "365/504" means that, of the 869 subjects within that sub-group, the majority (504) chose BCS while the remaining 365 chose mastectomy.

In our tree analysis, we initially selected a tree whose size was based on the results of repeated cross-validation, and we critically evaluated our initial choice by "burling" the tree, a process that examines alternative splitting rules at each node and assesses the performance of these alternatives against the originally chosen rule. In constructing the tree, we imposed a condition that all terminal nodes had to contain a minimum of 20 subjects. The results of our tree analysis show that tumor size is, by far, the greatest determinant of type of surgical treatment for breast cancer patients. Subjects with small tumors tend to choose BCS regardless of other factors, while, subjects with moderate to large tumors appear to behave differentially depending on a number of other factors. Notably, subjects with moderate to large tumors aged 60 years or over, or aged under 60 years with nodal involvement, or aged under 60 years with no nodal involvement but a lobular tumor tend to choose mastectomy. Correspondingly, subjects with moderate to large tumors aged under 60 years with no nodal involvement and ductal or other type tumor tend to choose BCS. Individually each relevant factor has a reasonably straightforward biological explanation relating it to type of surgical treatment, but it should be noted that the tree model yields *conditional *rather than unqualified information about the characteristics of subjects choosing the respective treatments. For example, factors such as nodal status, subject age or tumor histology do not appear to be critical factors in the choice for women with small tumors; rather, these factors become critical *given information about tumor size*. One of the benefits of tree models is their ability to communicate such conditional information simply, whereas logistic regression models simply label variables, including complex interaction terms, as either significant or not without providing information as to the structure of key conditional relationships more useful in a diagnostic context. The selected tree model is depicted in Figure 1.

**Figure 2:**
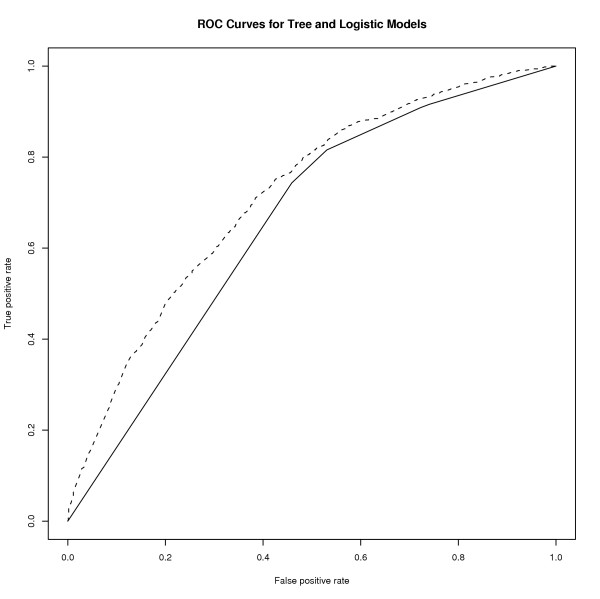
ROC curves for the fitted tree (solid line) and logistic regression (dotted line) models.

As a further comparison of the logistic and tree approaches to this analysis we produced an ROC curve for each model. The resulting curves are depicted in Figure 2. Based on these curves it appears the two models perform broadly competitively. The marginally better performance of the logistic model owes mainly to the fact that the selected logistic model has considerably more parameters than the selected tree model. However, these extra parameters mean that the slightly better performance of the logistic model from a sensitivity/specificity prospective comes at the expense of a loss of interpretability. Given the relative sizes of the tree model and the selected logistic regression model, the performance of the tree model is particularly good.

## Discussion

One of the benefits of tree models is that the output – a simple decision tree – is analogous to a diagnostic process with which medical professionals are familiar. As a result, the output of the tree model is generally easy to understand and summarize, even in the presence of significant two-way or even three-way interactions. This feature of trees allows the building of simple "profiles" of subjects who are predicted to prefer BCS over mastectomy and vice-versa. For example, patients with small tumors are likely to have BCS as their surgical treatment, while patients who have moderate to large sized tumors and are aged 60 years or older are likely to have mastectomy. Note that profiles of this kind derived from the tree are indicative rather than prescriptive, and that they describe tendencies, or broad patterns, rather than the behavior of particular individuals. Nevertheless, they are useful in characterising the patterns of patient behavior observed in the WACR data set, and in clarifying how the main factors identified in the analysis combine to influence patients' choices of surgical treatment for breast cancer. Tumor size is clearly the factor that dominates a patient's decision process, although a number of other factors appear relevant for patients with moderate to large sized tumors. The profiles paint rich but subtle pictures of patient behavior, indicating that patient decisions are often highly conditional rather than set by separate, individual rules for each relevant factor. This revelation would come as no surprise to doctors, as such a nested decision process would be entirely familiar to them in the context of advising their patients as to their most appropriate treatment option taking into account the patient's particular circumstances.

As with any analysis of complex data such as these, our tree analysis has limitations which must be acknowledged [15,21]. Firstly, tree models have a tendency to be quite variable or unstable, wherein a small change in the data can result in a quite different series of splits and hence a different tree model. This instability of tree models can make their interpretation somewhat open to question. While this problem is inherent to tree models, it is likely that the tree model produced in this paper is very stable at the first split on tumor size. The choice of this split was unequivocal as the improvement in goodness-of-fit (both from drop in deviance and misclassification standpoints) from splitting on size at this point was substantially larger than for any of the other explanatory variables. Nevertheless, the interpretation of splits low in the tree structure is somewhat more uncertain as alternative splits low in the tree may have produced a tree model with similar goodness-of-fit to that of the selected model. Secondly, due to the large number of statistical comparisons that are performed during the fitting of a tree, p-values are not particularly useful or interpretable for these models. This issue necessitates the use of cross-validation or the use of an independent data set to validate the tree model. A cross-validatory exploration revealed the selected tree model to be relatively stable, particularly so with regard to the first few splits. Thirdly, trees may not capture global linear relationships between the response and covariates because the tree must approximate linear effects with a series of binary splits [14]. These limitations illustrate that the easy interpretability and straightforward treatment of interactions characteristic of tree models compared to logistic regression models comes at some cost. It is important that these limitations be kept in mind when deciding whether classification trees are useful as an alternative to logistic regression in a study of this nature. Further, limitations imposed by the data must also be acknowledged. While data on tumor size was available, other relevant factors such as the size of the tumor relative to the size of breast and the degree of differentiation of the tumor were not available from this linked database.

Despite these limitations, our analysis offers some clear advantages over traditional approaches to analysing data such as these, and our findings are broadly useful for discovering which characteristics impact patients' choice of surgical treatment for breast cancer, and in estimating the extent to which each characteristic is important in the decision-making process.

## Conclusion

Classification trees perform as well as logistic regression as a predictor of patient choice, but are much easier to interpret for clinical use. The selected tree can inform clinicians' advice to patients, as well as to clarify complex interactions between covariates in predicting patient choice. In the context of this study of breast cancer in Western Australian patients, the tree model shows that tumor size is a major determinant of which surgical treatment patients choose, but that a variety of other factors, such as patient age, nodal status, and tumor histology, are also important in refining predictions of patient choice.

## Competing interests

The author(s) declare that they have no competing interests.

## Authors' contributions

RM obtained and cleaned the data, and also contributed to the statistical modelling and interpretation of the data. MM, TO and SR performed statistical analysis of the data and contributed to the interpretation and presentation of the analysis and discussion. All authors contributed to the drafting of the manuscript. All authors have read and approved the manuscript.

## Pre-publication history

The pre-publication history for this paper can be accessed here:



## Figures and Tables

**Table 1:** Summary statistics for subject characteristics, *N *= 2713. For categorical variables, the percentages of subjects in each category are presented. For continuous variables, five-number summaries are presented.

**Surgery**		**Aboriginality**		**Marital Status**	
Mastectomy	39.0%	No	99.2%	Married/De-facto	70.8%
Breast-Conserving Surgery	61.0%	Yes	0.8%	Otherwise	29.2%
**Method of Payment**		**Country of Birth**		**Area of Residence**	

Public, eligible for Medicare	49.4%	Australia	61.1%	Metropolitan	78.8%
Private, not insured	3.3%	Western Europe	28.2%	Rural	15.6%
Private, insured	45.8 %	Other	10.8%	Remote	5.6%
Ineligible for assistance	1.5%				
**Tumor Histology**		**Tumor Size (diameter in mm)**		**Patient Age (years)**	

Ductal	79.7%	Minimum	1 mm	Minimum	19 yrs
Lobular	12.1%	1^st ^Quartile	12 mm	1^st ^Quartile	47 yrs
Other (mixed/tubular/etc.)	8.1%	Median	18 mm	Median	56 yrs
		3^rd ^Quartile	25 mm	3^rd ^Quartile	65 yrs
		Maximum	100 mm	Maximum	95 yrs
**Nodal Staging Characteristics**		**Tumor Size Categories (T1/2/3)**		**Patient Age Categories**	

Node negative (N-)	58.8%	T1 (20 mm or less)	63.3%	Age under 40	8.2%
Node positive (N+) 1 to 3 nodes	26.9%	T2 (20 to 50 mm)	33.3%	Age 40 to 60	51.8%
Node positive (N+) 4 plus nodes	14.3%	T3 (exceeding 50 mm)	3.5%	Age 60 or over	40.0%

**Table 2:** Initial logistic regression model fit. Significant variables are denoted by ** (significant at 5%) or *** (significant at 1%).

Term	Coefficient	Standard Error	t-value	Significance (1% or 5%)
Intercept	-0.471	0.162	-2.905	***
*Tumor Size T1 (baseline)*				
Tumor Size T2	-0.500	0.045	-11.017	***
Tumor Size T3	-0.378	0.083	-4.576	***
*Subject Age under 40 (baseline)*				
Subject Age 40–60	-0.044	0.080	-0.547	
Subject Age ≥ 60	-0.144	0.035	-4.058	***
*Area of Residence – Metro (baseline)*				
Area of Residence – Rural	-0.210	0.058	-3.610	***
Area of Residence – Remote	-0.101	0.062	-1.638	
*Tumor Histology – Ductal (baseline)*				
Tumor Histology – Lobular	-0.151	0.064	-2.343	**
Tumor Histology – Other	0.175	0.056	3.112	***
*Lymph Node Negative (N-) (baseline)*				
Lymph Node Positive, 1–3 nodes	-0.216	0.049	-4.416	***
Lymph Node Negative, 4 or more nodes	-0.189	0.041	-4.625	***
*Country of birth – Australia/NZ (baseline)*				
Country of birth – Western Europe	0.039	0.050	0.797	
Country of birth – Other	-0.046	0.046	-1.011	
*Payment class – Public/Medicare (baseline)*				
Payment class – Private, not insured	0.045	0.122	0.366	
Payment class – Private, insured	0.024	0.046	0.518	
Payment class – Ineligible	-0.153	0.088	-1.735	
*Marital Status – Married/De-Factor (baseline)*				
Marital Status – Other	0.028	0.048	0.578	
